# Observations on Cupping

**Published:** 1809-12

**Authors:** 


### *To the Editors of the Medical and Physical Journal.*

GENTLEMEN,

HAVING read in your Journal, the request of the "Country Practitioner," and the answers of "Galen" and "Mr. Lyall," there is but little room left for my observations on the art of Cupping; I shall, therefore, merely hint at such remarks of your Correspondents as practice induces me to think less necessary, and add others unnoticed by them, but which I have found useful.

In

In all operations it is allowed by every one to be of the utmost importance to contract the period of the operation as much as possible, both on account of the patient and of the operator. 1st. The wiping of the glasses dry, as recommended by the Answerers, I shall show, will cause a lengthening in two ways; first, the act of wiping the glasses will take up some time; and secondly, during the time they are being wiped, a great portion of caloric will escape, and consequently cause the rarification of the air to be less perfect, and require a longer application of heat from the lamp to expel the air which has entered.

In order to render the process more expeditious, the water should be as hot as can conveniently be borne by the operator; and it will always be found sufficient to give the glass a shake, merely to throw out the larger drops; for, if the glass be filled with aqueous vapour, that will be rarified more quickly, *i. e.* with much less heat than the same bulk of colder air, and produce a vacuum equally perfect.

The next grand point in the operation is the obtaining the wished for quantity of blood. I allow the glasses to be applied to the parts before the scarificator is applied, in order to invite the blood to the parts; but the most effectual method is not suffering the blood to coagulate at all in the glasses, and removing them as soon as it begins to thicken; by this it is prevented from plugging up the orifices, and consequently the blood, on the application of other glasses, flows freely, the wounds being as it were fresh.

The rarification of the air is better performed by the spirit lamp, than by small pieces of bibulous paper dipped in spirit, or small filaments of tow; for two reasons, 1st. They will cause a *delay* in the process, as they require some preparation; and, 2dly, it makes the operation more clumsy, or not so neat, the pieces which are not burnt mixing with the blood. But the best method of rarifying the air with the greatest equality is by means of a small Argand's lamp. If the lower edge of the cupping glass be put about one-fourth or one-eighth of an inch below the top of the chimney, a greater heat may be applied, and the edges will still be sufficiently cool to be applied to the parts without pain. I am, &c.

